# Letter from Buffalo

**Published:** 1859-09

**Authors:** 


*Letter from Buffalo.*—We received the following letter from Buffalo, just too late for an August issue, and were therefore compelled to defer its publication till the present time. Our readers will see that medical matters are no more stirring in Buffalo during the summer months, than in other large cities. In New York, this inactivity is even more marked, most of the working men taking a few weeks' vacation during the warm weather, and the few who remain doing nothing in the way of unnecessary labor. We intend to keep our readers *au courant* in medical matters in Buffalo, as well as here, being in no wise desirous of losing our character as a Buffalo Journal, in which capacity it is our pride to think we have attained a dignified and respectable position. We will therefore publish letters from

Buffalo, from time to time, and keep ourselves informed of the medical news of our old home.

BUFFALO, July 21st, 1859.

*Dear Doctor :*—The Medical Society of our city met as usual on the first Tuesday in the month, but nothing of interest to the profession transpired. The alarming health that for more than a year has prevailed, may be one occasion for the lack of contributions sent in; and another reason, the warm weather has more or less effect in inducing a repugnance to undertaking any but necessary labor. The Health Physician's report of mortality for the month of June is published, and the total number of deaths is 118. I am tempted to call your readers' attention to one fact, that otherwise might be overlooked,—the large number of certificates returned monthly by sextons; for June, it is 42. By this you will perceive that about one third of the returns are certified to by undertakers. The question naturally arises, do all these people die without calling a medical man? I very much doubt it, and can only explain it on the theory of unwillingness of the sexton to go to the physician in attendance and get his certificate. They make one themselves, calling the death from fits, or consumption, or any other disease with the name of which they may be familiar. Hence you see the returns are very imperfect in a medical view, as basis for estimating with accuracy the deaths from any class of disease. The present City Physician, Dr. Strong, is doing a good thing in giving us all the facts in relation to who certify to cause of death, both where they die, and whether, if certified by physicians, they are regular M.D.s or not. Let us look at this matter for the first six months. From January to July, the total number of deaths has been 755; of this number, 222 have been certified to by sextons. For the months of February, March, April, and June, there were 93 deaths certified to by irregular physicians—an average of 23¼ per month. It is to be hoped that some remedy may be suggested to give us more accurate returns.

During this month, the new part of the Buffalo Lying-In Hospital has been brought into use. It is now quite a large building, and furnishes accommodations to two or three times the number heretofore enjoying its benefits. During the past year, patients have been admitted from many of the Western States, Canada, and our own State. Never but one fatal case of puerperal disease has occurred in it since its opening, now some four or five years since, and this was from fault of the patient. It is, as heretofore, under the medical care of Professor White, and the great success obtained in its wards is principally due to him.

Buffalo has lately received an addition to the ranks of its medical men in the persons of Dr. John Cronyn, who has heretofore resided at Fort Erie, in Canada, and Dr. Lothrop, of Boston, recently from Chicago. I hear little of surgical matters. I may mention, perhaps, the use of the écraseur, by Dr. White, in removing a cauliflower excrescence, the size of a fœtal-head, from the uterus, it being pulled down by Jobert's hooks, the operation consuming five minutes, &c., &c.

Dr. Storck has related a curious case to us: it is that of a German girl, fourteen years old, named Manner. She has confessed to having swallowed a number of pins from time to time; and, becoming unwell from one lodging in the throat, the Dr. was called in, and being unable to get it out, pushed it down, at the sameti me prescribing oleaginous substances, &c. The result was, the passage per anum of five needles and seventeen pins, twenty-two in all. The girl ate the pins, or swallowed them, that she might be sick and escape working out as a servant. We have seen the needles, and they present a corroded appearance, such as would be likely to be present after such a journey. Now for the popular phase of this. The above facts having been reported in the *Telegraph*, a German paper here, the *Demokrat*, also a German paper, denied its possibility, and accused the *Telegraph* of having been hoaxed, or endeavoring to do as much to the people. Considerable sparring back and forth has been the result; but I imagine the *Demokrat* editor is pretty well used up by the facts presented, in the shape of affidavits, medical precedents, &c., &c.

The great medical fact of the present moment here, is M. A. Schlosser, surgeon-chiropodist, who advertises to extract corns and bunions without pain; and, as usual, certificates are multiplied to establish his assertion. He fills whole columns of the daily papers with advertisements; he ought to thrive, to pay the printers. W. H. B.

